# Clinical and Neurobiological Aspects of TAO Kinase Family in Neurodevelopmental Disorders

**DOI:** 10.3389/fnmol.2021.655037

**Published:** 2021-03-24

**Authors:** Chun Hu, Pan Feng, Qian Yang, Lin Xiao

**Affiliations:** ^1^Key Laboratory of Brain, Cognition and Education Sciences, Ministry of Education, South China Normal University, Guangzhou, China; ^2^Institute for Brain Research and Rehabilitation, South China Normal University, Guangzhou, China

**Keywords:** TAO kinase, neurodevelopmental disorders, neuron, cytoskeleton, *de novo* mutations, therapy

## Abstract

Despite the complexity of neurodevelopmental disorders (NDDs), from their genotype to phenotype, in the last few decades substantial progress has been made in understanding their pathophysiology. Recent accumulating evidence shows the relevance of genetic variants in thousand and one (TAO) kinases as major contributors to several NDDs. Although it is well-known that TAO kinases are a highly conserved family of STE20 kinase and play important roles in multiple biological processes, the emerging roles of TAO kinases in neurodevelopment and NDDs have yet to be intensively discussed. In this review article, we summarize the potential roles of the TAO kinases based on structural and biochemical analyses, present the genetic data from clinical investigations, and assess the mechanistic link between the mutations of TAO kinases, neuropathology, and behavioral impairment in NDDs. We then offer potential perspectives from basic research to clinical therapies, which may contribute to fully understanding how TAO kinases are involved in NDDs.

## Introduction

Neurodevelopmental disorders (NDDs) are a group of heterogeneous conditions that fail to acquire multiple proper developmental milestones involved in cognitive, emotional, and psychomotor skills caused by abnormal changes in early brain development. NDDs include mainly autism spectrum disorder (ASD), intellectual disability (ID), developmental delay (DD), attention deficit hyperactivity disorder (ADHD), schizophrenia, and epilepsy and affect around 3–5% of children worldwide (Fitzgerald et al., 2015; Wilfert et al., 2017; Parenti et al., 2020; Hanly et al., 2021). In general, any possible factor that could disrupt tightly programmed and coordinated events will eventually result in NDDs (Parenti et al., 2020). Although NDDs have been intensively investigated for many years, from the bench to the clinic, little is known about their causal risk factors and/or genes and fundamental neurobiology due to their complexities from their genotypes to phenotypes (Sullivan and Geschwind, 2019; Moyses-Oliveira et al., 2020; Parenti et al., 2020). One of the most productive areas of NDDs research, however, lies in human genetics. The combined genome-wide association study (GWAS; Visscher et al., 2017) and next generation techniques based on whole genome and exome sequencing (WGS and WES; Sanders et al., 2018; Coe et al., 2019; Satterstrom et al., 2020), have identified quite a few of susceptive genes for developing NDDs, which provides further opportunities to study the underlying mechanisms of how genetic mutations contribute to specific NDDs. Thus, to identify reproducible high risk genes and to reveal their underlying neurobiological mechanisms in molecular, cellular, and circuit-levels is critical for developing personalized treatments for NDDs (Krystal and State, 2014).

**Figure 1:**
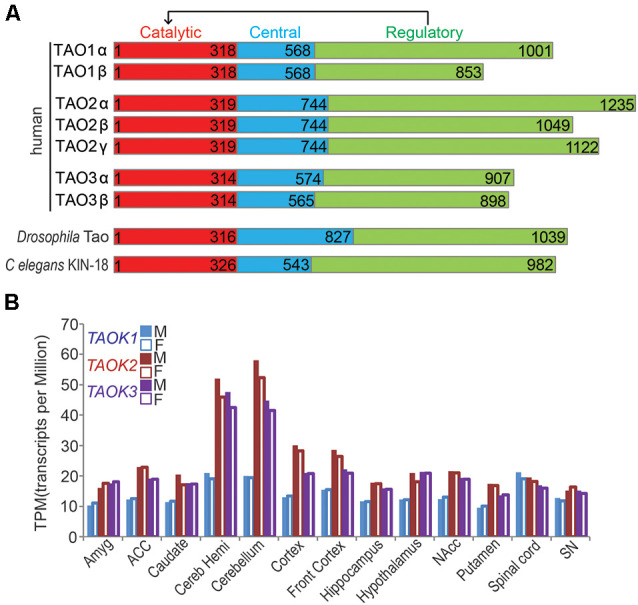
Thousand and one (TAO) kinases are highly conserved. **(A)** Schematic structure of conserved TAO kinase family is shown. A typical protein structure of TAO kinase includes a catalytic domain, a central domain, and a regulatory domain, which could regulate catalytic activity. In humans, three genes of TAO kinases encoding TAO1, TAO2, and TAO3 are present, each of them has two isoforms (α and β, except TAO2 has the third putative one γ isoform) which may take different biological functions. Only a single Tao gene is encoded in *Drosophila melanogaster* (*CG14217*, *Tao*) and *C. elegans* (*kin-18*). **(B)** The relative expression levels (TPM) of human TAO kinases in different areas of the central nervous system (CNS) are shown. The bar represents the median value from RNA sequencing. Data source: the Genotype-Tissue Expression (GTEx) project. M, male; F, female.

TAO (Thousand and one) kinase family belongs to the STE20 group kinases and consists of three genes in vertebrates including *TAOK1*, *TAOK2*, and *TAOK3* that encode TAO1, TAO2, and TAO3, respectively (Dan et al., 2001; Miller et al., 2019). They have relatively close homologs in several invertebrate species like *C. elegans* and *Drosophila*, which structurally contain three conserved domains including the kinase domain, central domain and regulatory domain (Figure 1A). TAO kinases were reported to play multifunctional roles in many molecular and cellular events by interacting with MAPK cascade, MST family kinases, the cytoskeleton, and apoptosis-associated proteins (Duan et al., 2020). Therefore, it is not surprisingly that TAO kinases could regulate neuronal survival (Wakabayashi et al., 2005; Wu and Wang, 2008; Li et al., 2019) and development (Yasuda et al., 2007; de Anda et al., 2012; Ultanir et al., 2014; Yadav et al., 2017; Richter et al., 2018; Dulovic-Mahlow et al., 2019) in the nervous system. Although there is plenty of evidence suggesting that TAO kinases likely contribute to NDDs, the direct association between TAO kinases and NDDs has not been fully discovered until recently.

The original discovery of two genomic deletions in *TAOK1* is from patients with developmental delay (nsv1062993; Cooper et al., 2011), microcephaly, and seizures (Decipher 250045; Decipher database; Xie et al., 2016). In addition, Xie et al. (2016) also identified a 17q11.2 microdeletion that covers *TAOK1* in a patient with developmental delay and postnatal microcephaly, implying the copy number variants (CNVs) of *TAOK1* is likely involved in NDDs. Following the exome sequencing of over 4,200 individuals with developmental disorders, researchers found four missense *de novo* mutations in *TAOK1* that likely contribute to those developmental disorders (Deciphering Developmental Disorders Study, 2017). Subsequently, a WES based analysis further identified eight *de novo* mutations in *TAOK1* from eight patients with different NDDs including speech and language development and/or motor development delay, muscular hypotonia, macrocephaly, seizures, and intellectual disability (Dulovic-Mahlow et al., 2019). In addition, a total 23 patients with NDDs who carry different monogenic *TAOK1* variants (20 individuals) or a microdeletion covering *TAOK1* (three individuals) were discovered in a recent study (Woerden et al., 2021). These studies provide solid evidence that both pathogenic CNVs and single point mutations that disrupt *TAOK1* could have a deleterious effect that contributes to NDDs.

*TAOK2* is supposed to be a susceptive gene of NDDs and is based on the findings that 16p11.2 microdeletion or microduplication contributes to multiple NDDs (Weiss et al., 2008; McCarthy et al., 2009; Steinman et al., 2016). Human *TAOK2* is one of the 30 genes located in the microdeletion or microduplication region of 16p11.2. By performing whole-genome sequencing (WGS) of families with ASD, one frame shift deletion of *TAOK2* was identified (Yuen et al., 2017). This was further confirmed in a following study (Richter et al., 2018), in which two other more *de novo* mutations of *TAOK2* was identified in ASD patients (Richter et al., 2018).

Compared to those repeatedly identified *TAOK1* and *TAOK2* *de novo* mutations in NDDs, less attention was paid to the *TAOK3* gene. A previous analysis of *de novo* CNVs by WGS in individuals suffering from bipolar disorder and schizophrenia showed that a microdeletion that affects *TAOK3* (and *PEBP1*) is present in schizophrenia patient (Malhotra et al., 2011) and *TAOK3* (but not *PEBP1*) was further confirmed in a GWAS analysis (Gilman et al., 2012), suggesting that *TAOK3* alone may contribute to NDDs, at least in schizophrenia. The direct evidence for monogenic *TAOK3* mutations contributing to NDDs is from a comprehensive study with WES, which systematically analyzed over 2,500 autistic children and 1,900 unaffected siblings and the parents of each family and eventually identified two validated *de novo* mutations of *TAOK3* in ASD patients (Iossifov et al., 2014). It would be beneficial to identify more monogenic mutations of *TAOK3* in ASD or other NDDs in future studies.

Because each individual TAO kinase was reported to be associated with NDDs, it is important to investigate and obtain new insights into their biological mechanisms in neurodevelopment to confirm their potential roles in NDDs. Thus, despite the progress made in both basic and clinical research of TAO kinases related NDDs in the last few years, the systematic summary of these findings are lacking. In this review, we will introduce TAO kinases functions in general, summarize the *de novo* mutations that contribute to multiple NDDs, and discuss the possible underlying molecular and cellular mechanisms. Lastly, we propose future directions of an intensive understanding of TAO kinases in neurodevelopment and NDDs, which might be critical in the development of potential and precise treatment of specific NDDs.

## TAO Kinases in General

### TAO Kinase Structure and Expression Patterns in the Central Nervous System (CNS)

The TAO (Thousand and one) kinase subfamily is part of the larger STE20 (sterile 20) kinase family, a diverse group of serine-threonine kinases that participate in a variety of signaling pathways (Dan et al., 2001). The TAO kinase family is highly conserved, and has three members in mammals including *TAOK1*/*PSK2*/*MARKK* (Hutchison et al., 1998), *TAOK2*/*PSK1* (Moore et al., 2000), and *TAOK3*/*JIK* (Tassi et al., 1999), but just a single representative TAO kinase in *C. elegans* (*Kin-18*; Berman et al., 2001; Spiga et al., 2013; Yin et al., 2016) and *Drosophila melanogaster* (*Tao*); (Liu et al., 2010). Structurally, all members of TAO kinases contain three domains including the N-terminal catalytic domain, the central domain, and the C-terminal regulatory domain which regulates catalytic activity (Figure 1A). Among these domains, the catalytic domains of all TAO kinase members are extremely conserved, but the regulatory domains may determine their distinct functions. In addition, each member of mammalian TAO kinases has two isoforms (α and β) except for TAO2, which has a putative third one (TAO2γ) which is mainly distinguished by the regulatory domain (Figure 1A), suggesting a distinct biological function of the different isoforms. Thus, it was reported that the two isoforms (α and β) of TAO2 play different roles in dendritic and dendritic spine development (Yasuda et al., 2007; Richter et al., 2018), the possible isoform-dependent functions in other members should be further explored.

TAO kinases are ubiquitously expressed without obvious tissue specificity based on mRNA transcription and protein translational levels (Duan et al., 2020), although it was reported that *TAOK1* and *TAOK2* are highly enriched in the brain by Northern blot detection (Hutchison et al., 1998). In the CNS, TAO kinases are widely distributed in all brain regions and the spinal cord without gender differences^1^ (Figure 1B). In addition, TAO kinases are modestly expressed but *TAOK2* and *TAOK3* showed strong expression in the cerebellum (including cerebellar hemisphere) and cortex (including front cortex), implying that the importance of these brain regions are critical for TAO kinase related NDDs.

### The General Functions of TAO Kinases

**Figure 2:**
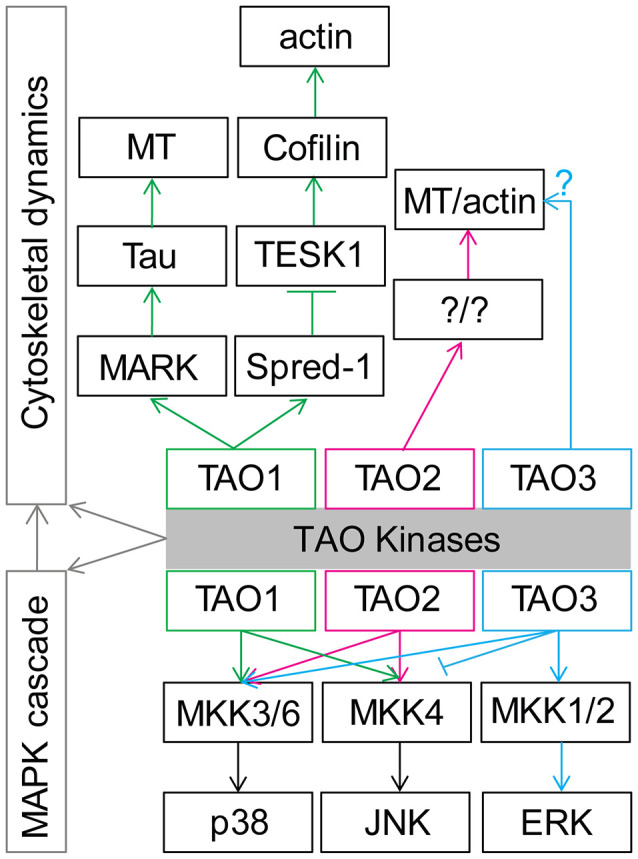
General functions of TAO kinases in regulation of cytoskeletal dynamics and mitogen activated protein kinase (MAPK) cascade cytoskeletal dynamics. See the main text for details. Note that TAO1 and TAO2 share similar functions, although TAO1 preferentially activates p38 and TAO2 preferentially activates JNK. Importantly, the possible distinct function between TAO3 and TAO1/2 should be further examined.

As TAO kinases belong to the STE20 kinase family, the original functions of TAO kinase are supposed to act as upstream regulators of mitogen activated protein kinases (MAPKs). Upon extracellular stimulations, conventional MAPK cascades including the ERK1/2, p38 MAPKα, β, δ, and γ, and JNK1/2/3 pathways are selectively activated to integrate, amplify, and regulate signal transduction and eventually affect multiple biological processes including cell proliferation, differentiation, migration, and apoptosis (Johnson and Lapadat, 2002; Kyosseva, 2004; Cargnello and Roux, 2011). TAO1 and TAO2 have a lot in common regarding activating p38 MAPK (Hutchison et al., 1998; Chen et al., 1999) and the JNK cascade (Moore et al., 2000; Zihni et al., 2006). TAO3 shows a relatively distinct function that activates ERKs (Zhang et al., 2000) and p38 MAPK but inhibits the JNK cascade (Tassi et al., 1999). It should be noted that TAO3 may inhibit p38 kinases in certain conditions (Tassi et al., 1999). Thus, the TAO kinases family is a central regulator in controlling MAPKs cascades and may be involved in multiple biological processes (Figure 2).

In addition, TAO1 is also known as MARKK (microtubule affinity regulating kinase) due to its ability to phosphorylate MARK, which can further affect microtubule (MT) arrangement by modulating tau (Giacomini et al., 2018) and other related microtubule-associated proteins (MAPs; Drewes et al., 1997), indicating the functional role of TAO1 in regulating MT dynamics. Further research found that TAO1 could interact with Spred-1 to inhibit TESK1, which could modulate actin dynamics by activating Cofilin (Moore et al., 2000). At the same time, TAO2 was also reported to modulate actin filament and MT rearrangement in cultured 3T3 cells (Moore et al., 2000; Mitsopoulos et al., 2003). Although direct evidence remains is scarce, TAO3 might also play a role in the regulation of cytoskeletal dynamics due to the fact that it has been reported that this kinase modulates the JNK signaling (Kapfhamer et al., 2012; Zeke et al., 2016). It is therefore not surprising that *Drosophila* Tao, the only homolog of mammalian Tao kinases, affects actin and MT dynamics in cultured *Drosophila* S2 cells (Liu et al., 2010; Pflanz et al., 2015) and primordial germ cells from developing embryos (Pflanz et al., 2015). It should be noted that the MAPK cascade signaling pathway has a tight connection with cytoskeletal dynamics (Šamaj et al., 2004; Komis et al., 2011). Therefore, TAO kinases’ functions may be tightly involved in cytoskeletal dynamics regulation (Figure 2).

## Clinical Association Between TAO Kinases and NDDs

Because TAO kinases is intensively involved in regulating cytoskeletal dynamics, which is required for nearly all processes of normal neuronal and glia development involving cellular survival, migration, polarity, differentiation and plasticity (Conde and Cáceres, 2009; Hoogenraad and Bradke, 2009; Kapitein and Hoogenraad, 2015; Konietzny et al., 2017; Weigel et al., 2020), they are always speculated to be risk genes for NDDs. However, this reasonable speculation has not, until recently, been proven to be correct, benefiting from powerful next- generation sequencing techniques and the establishment of well-organized cohorts [e.g., Simons Simplex Collection (SSC) cohort; Fischbach and Lord, 2010] and networks [e.g., the Deciphering Developmental Disorders (DDD) network; Firth and Wright, 2011] of investigators.

### TAOK1

A rare *de novo* microdeletion at 17q11.2, which covers *TAOK1*, was identified in a patient with a developmental delay and postnatal microcephaly (Xie et al., 2016), suggesting a possible role of *TAOK1* to contribute to NDDs. Two genomic microdeletions in the *TAOK1* genome (without affecting other genes) in patients with developmental delay (nsv1062993; Cooper et al., 2011), microcephaly, and seizures (Decipher #250045, Decipher database) was reported. Importantly, the deletion nsv1062993 completely overlaps the CNV of #250045, and the two partially overlap the *TAOK1* gene. It is assumed that the *TAOK1* gene plays a pivotal role in the phenotype of patients (Xie et al., 2016). In addition, *TAOK1* was predicted to have a haploinsufficiency score less than 10, suggesting one copy deletion of *TAOK1* could cause clinical consequences (Xie et al., 2016). Followed by an integrated meta-analysis that combines *de novo* mutations from exome sequencing data and CNV morbidity data, *TAOK1* with a missense mutation was identified as a candidate for NDDs (Coe et al., 2019). Furthermore, in a large scale whole exome screening (WES) of patients with autism, *TAOK1* was identified as one of over 100 putative autism spectrum disorder (ASD) associated genes (Satterstrom et al., 2020). In addition, *TAOK1* *de novo* mutations were identified from eight children with associated NDDs including delayed speech and language development (5/8), autism (2/8), intellectual deficiency (4/8), macrocephaly (3/8), and motor development delay (6/8; Dulovic-Mahlow et al., 2019). Interestingly, half of these *de novo* mutations locate in the catalytic domain, one truncated mutation is present in the central domain, and the other three are truncated or frameshifted mutations in the regulatory domain. Although the precise functional analysis of these mutations is still lacking, the blood and fibroblast line derived from a patient carrying a variant (c.2366_2367insC) was analyzed. It was found that this variant could decrease cDNA (Sanger sequencing), mRNA (quantitative real time PCR), and protein (western blot) levels of *TAOK1*. In addition, the mutated mRNA could be stabilized by cycloheximide treatment indicating that nonsense-mediated mRNA decay could explain the reduced abundance of the mutant allele (Dulovic-Mahlow et al., 2019). Interestingly, a recent study collected a cohort of 23 individuals with NDDs from a collaboration facilitated by GeneMatcher (Sobreira et al., 2015) with multiple *de novo* variants in *TAOK1*, among which 20 individuals had an intragenic *TAOK1* variant and three patients had a chromosomal deletion including *TAOK1* (Woerden et al., 2021). Importantly, this study tested several variants with functional assays in a mouse model showing that two variants (c.500T>G and c.943C>T) may act with dominant negative (DN) functions and one variant (c.1643T>C) may have a loss of function (LOF) effect. Thus, these studies provide clear evidence that dysfunction of *TAOK1* with either CNVs or mutations with DN or LOF effect will result in NDDs.

### TAOK2

*TAOK2* is highlighted as a candidate risk gene for NDDs because previous reports showing that microdeletion and microduplication of the 16p11.2 genetic locus are associated with ASDs or schizophrenia (Weiss et al., 2008; McCarthy et al., 2009; Zheng et al., 2013). *TAOK2* is one of the 30 genes that are located in the 16p11.2 region. Recently, more psychiatric features like speech/language impairments, intellectual disability, motor/developmental delay, microcephaly and macrocephaly have been identified in patients with 16p11.2 microdeletion and microduplication (Steinman et al., 2016; Rein and Yan, 2020). Although it has been long been proposed that disruption of monogenic *TAOK2* function might result in NDDs, the clear clinical evidence has only been presented recently. By performing whole-genome sequencing (WGS) of families with ASD, one frame shift deletion of *TAOK2* was identified (Yuen et al., 2017). This was further confirmed in a following study (Richter et al., 2018). Richter et al. (2018) combined WGS and WES to examine over 2,600 families with ASDs and identified 24 different variants in *TAOK2*, including the frame shift deletion identified by Yuen et al. (2017). They further characterized that three of these variants are known to be *de novo* including a missense mutation in the catalytic domain (A135P), a frameshift deletion resulting in truncation (P1022*) in the regulatory domain, and a *de novo* splice site variant (c.563 + 12_563 + 15del) predicted to cause intron seven retention. Among these three *de novo* mutations, A135P mutation results in lower levels of both phosphorylated and total TAO2 but the P1022* mutation does not affect TAO2 levels in patients with derived lymphoblastoid cells. Combined with more biochemical analysis, A135P and P1022* were confirmed to abolish (LOF) and enhance (GOF) kinase activity, respectively. The impact of the *de novo* splice site variant (c.563 + 12_563 + 15del), however, is still unknown and requires further characterization (Richter et al., 2018). It should be noted that, patients carrying these *de novo* mutations showed typical ASDs symptoms and also have a certain degree of disability or delay in language and speech development, implying the broad function of *TAOK2* in neurodevelopment.

Although *TAOK2* contribution to 16p11.2 CNVs pathophysiology is quite convincing, it should be noted that it is *TAOK2* and other genes (e.g., *MAPK3*, *SEZ6L2* and *KCTD13*) that act collectively to develop much complex and diverse 16p11.2 CNVs phenotypes (Krishnan et al., 2016), compared to individual gene mutations (Richter et al., 2018; Rein and Yan, 2020). In this review, we focus on *TAOK2* function only since 16p11.2 CNVs related NDDs have recently been reviewed (Rein and Yan, 2020) and is also out of scope of the current study.

### TAOK3

Unlike *TAOK1* and *TAOK2*, studies on the association of *TAOK3* with NDDs is relatively scarce. Several GWAS based studies indicate that *TAOK3* is likely involved in NDDs. A study with a rare CNVs analysis by WGS suggested that a *de novo* deletion that affects *TAOK3* (and *PEBP1*) may contribute to schizophrenia (Malhotra et al., 2011) and *TAOK3* (but not *PEBP1*) was further confirmed in a GWAS analysis (Gilman et al., 2012), suggesting LOF of monogenic *TAOK3* may contribute to NDDs, at least in schizophrenia. In another GWAS study, *TAOK3* was identified as a genetic predisposition to loneliness (Abdellaoui et al., 2019), a status that accompanies NDDs (Kwan et al., 2020; Papagavriel et al., 2020). In addition, two independent GWAS analyses showed that *TAOK3* is related to high opioid requirement for patients with advanced cancer pain (Gutteridge et al., 2018) and morphine requirement for postoperative pain in a retrospective pediatric day surgery population (Cook-Sather et al., 2014), suggesting a functional role of *TAOK3* in controlling pain under certain conditions. Since the impairment of sensory perception/processing is highly associated with NDDs, like autism (Robertson and Baron-Cohen, 2017), the possible function of *TAOK3* in NDDs is further implied. Except for GWAS studies, in a genome-wide-association meta-analysis (GWAMA), *TAOK3* was also identified as a susceptive gene for depression (Baselmans et al., 2019). Overall, those studies suggest a possible role of *TAOK3* in NDDs.

The direct evidence for monogenic *TAOK3* links to NDDs is based on a large scale WES that compared and analyzed over 2,500 affected ASD children and 1,900 unaffected siblings and the parents of each family (Iossifov et al., 2014). In this systematical study, two novel missense *de novo* mutations *TAOK3* (c.1495A>G and c.1894C>T) were identified and validated (Iossifov et al., 2014; Table 1). These two missense mutations encode protein TAO3 with pT199A and pR632W, which are located in the central and regulatory domain, respectively. Although they are likely deleterious mutations as predicted in the gnomAD database, it should be noted that to determine whether these two mutations in *TAOK3* are pathogenic or not requires a detailed functional analysis and more clinical samples. Thus, it would be better elucidated if more deleterious mutations of *TAOK3* in ASD or other NDDs patients are identified and validated in future studies.

**Table 1:** A selected list of *de novo* mutations between thousand and one (TAO) kinases and neurodevelopmental disorders (NDDs).

	Variants	Amino acid change	Monogenic	Data type	Diagnosis	Reference/source
*TAOK1* (NM_020791.2)	c.50A>G	E17G	Yes	WES	Delayed speech and language; ID; ADHD	Dulovic-Mahlow et al. (2019)
(NM_020791.2)	c.332C>T	S111F	Yes	WES	Delayed speech and language; ID; ASD	Dulovic-Mahlow et al. (2019)
(NM_020791.2)	c.892A>G	K298E	Yes	WES	Delayed speech and language; Macrocephaly; ASD	Dulovic-Mahlow et al. (2019)
(NM_020791.2)	c.914A>C	D305A	Yes	WES	ID	Dulovic-Mahlow et al. (2019)
(NM_020791.2)	c.1630C>T	Q554*	Yes	WES	ADHD	Dulovic-Mahlow et al. (2019)
(NM_020791.2)	c.2341G>T	E781*	Yes	WES	ID; Macrocephaly	Dulovic-Mahlow et al. (2019)
(NM_020791.2)	c.2366_2367insC	L790Ffs*3	Yes	WES	Delayed speech and language; ID; ASD	Dulovic-Mahlow et al. (2019)
(NM_020791.2)	c.2488G>T	E830*	Yes	WES	Delayed speech and language	Dulovic-Mahlow et al. (2019)
(NM_020791.2)	c.656C>T	A219V	Yes	WES	Developmental disorders	Deciphering Developmental Disorders Study (2017)
(NM_020791.2)	c.500T>G	L167R	Yes	WES	Developmental disorders	Deciphering Developmental Disorders Study (2017) and Woerden et al. (2021)
(NM_020791.2)	c.70C>A	P24T	Yes	WES	Developmental disorders	Deciphering Developmental Disorders Study (2017)
(NM_020791.2)	c.865G>A	V289M	Yes	WES	Developmental disorders	Deciphering Developmental Disorders Study (2017)
(NM_020791.2)	c.943C>T	L315F	Yes	WES	NDDs	Woerden et al. (2021)
(NM_020791.2)	c.1643T>C	L548P	Yes	WES	NDDs	Woerden et al. (2021)
(NM_020791.2)	Partial deletion chr17:277713 42-27809321	P?	Yes	WGS	Microcephaly and seizures	Decipher database (Xie et al., 2016)
(NM_020791.2)	Partial deletion chr17:277305 73-27802767	P?	Yes	WGS	Developmental delay	Cooper et al. (2011) and Xie et al. (2016)
17q11.2 (covers *TAOK1*)	Deletion		No	WGS	Developmental delay and postnatal microcephaly	Xie et al. (2016)
17q11.2 (covers *TAOK1*)	Deletion		No	WES	NDD	Woerden et al. (2021)
*TAOK2* (NM_016151)	c.403G>C	A135P	Yes	WES, WGS	ASD	Richter et al. (2018)
(NM_004783)	c.3057_3081del	P1022*	Yes	WES, WGS	ASD; Delayed language	Yuen et al. (2017) and Richter et al. (2018)
(NC_000016.9)	c.563 + 12_ 563 + 15	P?	Yes	WES, WGS	ASD	Richter et al. (2018)
16p11.2 (covers *TaoK2*)	Deletion		No	WGS	ASD	Weiss et al. (2008)
16p11.2 (covers *TaoK2*)	Duplication		No	WGS	ASD, Schizophrenia	Weiss et al. (2008) and McCarthy et al. (2009)
TAOK3	Deletion		No	WGS, GWAS	Schizophrenia	Malhotra et al. (2011) and Gilman et al. (2012)
(NM_016281.3)	c.1495A>G	T499A	Yes	WES	ASD	Iossifov et al. (2014)
(NM_016281.3)	c.1894C>T	A632T	Yes	WES	ASD	Iossifov et al. (2014)

CNVs of 17q11.2 and 16p11.2 locus are shown in the list because they cover *TAOK1* and *TAOK2*, respectively. In total, 20 point mutations of *TAOK1* were identified (Woerden et al., 2021) and three of the *de novo* mutations showing functional changes were selected. Note: the gene with the affected mRNA accession no. is indicated if available in the original studies/database. The individual variant is labeled; deletion or duplication means the whole gene is deleted or duplicated in the genomic fragment while partial deletion means the deletion fragment is located in the affected gene, without affecting other genes. Amino acid change: * indicates the stop codon. WGS, whole genome sequencing; WES, whole exome sequencing; GWAS, genome-wide associated study.

## Neuronal Mechanisms of TAO Kinases in Neurodevelopment and NDDs

**Figure 3:**
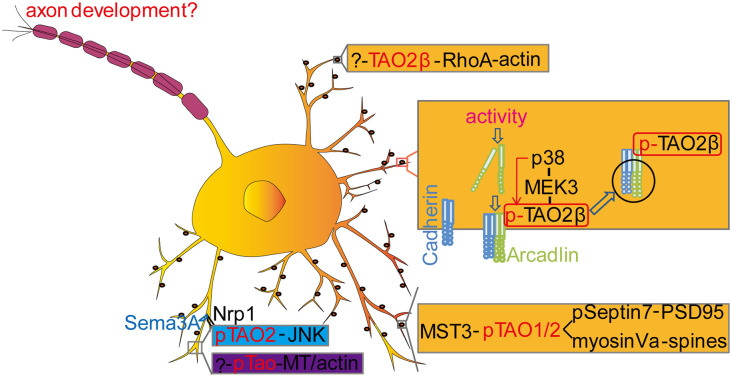
Molecular mechanisms of Tao kinases in neurodevelopment. TAO2 could regulate dendrite development (blue labeled) and spine development (yellow labeled) with multiple signaling pathways. In *Drosophila*, Tao kinase regulated dendritic development is also indicated with an unknown upstream pathway [?-pTao-Microtubule (MT)/actin] (purple labeled). In addition, TAO2 functions downstream of BDNF to activate the JNK signaling cascade to regulate interneuron maturation during development. However, whether TAO kinases contribute to axonal development and the underlying mechanisms remains to be determined. See details in the main text.

TAO kinases mutations that caused multiple NDDs could be a result of neurological and/or non-neurological impairments. So far, however, the non-neurological roles of TAO kinases in neurodevelopment and NDDs has not been reported. We will focus on the neurological aspect and summarize the accumulated data that implies an essential role of TAO kinases in regulating neuronal development (Figure 3), survival, and maturation.

### TAO Kinases Regulate Dendrite Development

Although TAO kinases at the mRNA level are widely expressed in adult human brains (Figure 1B), TAO2 is preferentially highly expressed in the intermediate zone and the cortical plate of the developing cortex (E18) in mice (de Anda et al., 2012). Interestingly, TAO2α is expressed from early embryonic stages (E10) to adults but TAO2β is only detectable at late stage (from E19) in the mouse brain cortex. Immunostaining with mouse cortical neurons (E17) and cultured 2 days *in vitro*, TAO2 is found to locate in the growth cone where actin is enriched and its active form pTAO2 was found to localize at the neurite shaft where MT is accumulated (de Anda et al., 2012), suggesting a possible role of TAO2 in regulating neuronal cytoskeletal dynamics. Knockdown of *Taok2* decreases basal dendrite arborization and callosal axon projection in the developing cortex (de Anda et al., 2012). Moreover, TAO2 could act downstream of the secreted guidance cue Semaphorin 3A (Sema3A) by interacting with its receptor Neuropilin 1 (Nrp1) to activate the JNK signaling pathway (de Anda et al., 2012).

To further characterize the neurodevelopmental functions of *Taok2* in mice, Richter et al. (2018) systematically analyze the neurological phenotypes in *Taok2* knockout (KO) mice, which were previously found to control behavioral response to ethanol in mice (Kapfhamer et al., 2013). Magnetic resonance imaging (MRI) of fixed 8- to 10-week-old mouse brains of *Taok2* KO and HET (KO/+) brains found that their absolute brain volumes were significantly enlarged compared with wild type (WT) mice but have a relative decrease in the somatosensory cortex and corpus callosum in a gene dose dependent way. Behavioral analysis indicated that the impairments on cognition, anxiety, and social interaction in *Taok2* KO mice were consistent with previous clinical studies of ASD patients (Hazlett et al., 2005; Freitag et al., 2009; Hardan et al., 2009; Vaccarino and Smith, 2009; Lai et al., 2014; Sacco et al., 2015). By examining the dendritic morphology in the prefrontal cortex, they found that *Taok2* KO and HET mice showed a decrease in basal dendritic complexity, which is comparable to the previous *in vitro* study (de Anda et al., 2012).

A recent study also clearly showed that overexpression (OE) of human TAO1 and its several NDDs linked variants in cultured primary hippocampal neurons from mice, could significantly reduce dendritic arborization and length (Woerden et al., 2021) although the detailed mechanisms remain to be further explored. Thus, to obtain more physiological evidence, further exploration of its function in neuronal/dendrite development, by generating *Taok1* KO mice, is required.

We recently also found that *Drosophila Tao* could significantly affect peripheral dendritic development of dendritic arborization (da) neurons at whole larval developmental stages and in adults (Hu et al., 2020). da sensory neurons are a well-characterized model system to study neuronal development and functions (Grueber et al., 2002; Williams and Truman, 2004; Shimono et al., 2009; Jan and Jan, 2010; Im and Galko, 2012; Copf, 2015). Using this model, many of the NDDs related genes and their molecular mechanisms have been confirmed and identified (Gatto and Broadie, 2011; Coll-Tane et al., 2019). We found that *Drosophila* Tao is expressed in all da sensory neurons and its active form pTao is discretely distributed along dendrites, suggesting a local function of *Drosophila* Tao in regulation of MT and/or actin to affect dendritic development. Loss of *Drosophila Tao* increases dendritic complexity and MTs dynamic of all da sensory neurons *in vivo* in a developmental stage dependent way (Hu et al., 2020), which is the opposite to the phenotype of neocortex neurons from an *in vitro* culture system (de Anda et al., 2012) or *TAOK2* KO mice (Richter et al., 2018). Interestingly, this increased dendritic complexity phenotype could be fully rescued by wild type human TAO2 but not by the ASD linked LOF mutation (A135P), suggesting a conserved function of *Drosophila Tao* and TAO2; but its readout varies in different model systems (King and Heberlein, 2011). Moreover, we also found that disruption of *Drosophila Tao* in adult sensory neurons caused dendritic over-branching and resulted in impairment of social behaviors, which further confirmed the previous observations that sensory perception is critical for developing ASDs in a mouse model (Orefice et al., 2016, 2019).

Altogether, these different *in vitro* and *in vivo* models clearly showed a critical and complex role of TAO kinases in regulating dendrite development which then affects normal brain functions.

### TAO Kinases Regulate Spine/Synapse Development

Both dendrite arborization and synapse formation are critical for wiring the neural circuitry and establishing normal neural functions (Benson et al., 2001; Jan and Jan, 2001; McAllister, 2007; Colón-Ramos, 2009; Batool et al., 2019). Yadav et al. found that TAO2 localizes to dendritic spines and is required for synaptic maturation in a kinase activity dependent way (Yadav et al., 2017). Combined with an elegant chemical-genetic method and mass spectrometry, the authors identified several candidate substrates of TAO2 including Septin6, Septin7, HADC6, Bai1, Caskin1, CEP170, and MST3, and only Septin7 was further confirmed in a subsequent functional analysis. Septin7 is a GTP-binding protein (Neubauer and Zieger, 2017) that regulates actin (Hu et al., 2012; Mavrakis et al., 2014) and MT remodeling (Bowen et al., 2011; Hu et al., 2012) to control axon/dendrite branching and spine morphology (Xie et al., 2007; Hu et al., 2012). It was found that TAO2 could directly phosphorylate Septin7 and lead to its trafficking to the dendritic spine where it could associate with and immobilize the synapse scaffolding protein PSD95 to promote spine synapse maturation (Yadav et al., 2017). In addition, a peptide pull-down method to identify binding proteins in neuronal lysates labeled by stable isotope labeling by amino acids in culture (SILAC; Zhang et al., 2011; Deng et al., 2019) was employed to identify possible binding components of TAO1/2, showing that Myosin Va could interact with TAO1/2 in a phosphorylation dependent manner. Moreover, endogenous Myosin Va could bind endogenous TAO1 and be phosphorylated by TAO1/2 in neurons (Ultanir et al., 2014). Myosin Va is a motor protein in charge of the intracellular transport of vesicles, organelles, and protein complexes along the actin filaments (Harrington and Rodgers, 1984; Masters et al., 2016; Guhathakurta et al., 2018; Lombardo et al., 2019) and affects microtubule based transport when recruited on the same cargo with the microtubule motor kinesin (Kapitein et al., 2013), suggesting a role of Myosin Va in promoting synaptic formation/maturation when localized in the spine (Ultanir et al., 2014). In addition, the authors found that TAO1/2 could be phosphorylated by mammalian sterile 20 (Ste20)-like kinase 3 (MST3), a homolog to *Drosophila Hippo* (Harvey et al., 2003). Interestingly, in contrast to the downstream of mammalian Tao Kinase to MST3, it seems *Drosophila* Tao is an upstream signaling component activating Hippo to regulate tissue growth (Boggiano et al., 2011; Poon et al., 2011, 2018; Huang et al., 2014; Chung et al., 2016). However, whether *Drosophila* Tao could phosphorylate Hippo to regulate synaptic growth needs to be further explored.

In another study, it was shown that TAO2β instead of TAO2α is essential for activity-induced dendritic spine formation (Yasuda et al., 2007), which rises a possibility that TAO kinases function in an isoform-dependent way. Electroconvulsive or other excitatory stimuli in cultured hippocampal neurons triggers a protocadherin arcadlin that stimulates TAO2β specifically, which in turn activates p38 MAPK through MEK3, resulting in the endocytosis of N-cadherin and the decrease in spine numbers (Yasuda et al., 2007; Sun and Xie, 2012), suggesting an isoform specific function of TAO kinases in spine development. This finding was further confirmed in the *Taok2* KO mouse model (Richter et al., 2018). It was shown that dendritic spines in hippocampal neurons from *Taok2* KO mice were dramatically decreased compared to WT, possibly by directly affecting the RhoA activity, a kinase which is also preferentially combined to TAO2β and is highly involved in the regulation of spine mobility. Those data indicate that the C-terminal of distinct TAO kinases is critical for their unique physiological functions.

Using *Drosophila* (neuron-muscle junction) NMJ as a classic system for studying synaptic development (Broadie and Bate, 1995; Menon et al., 2013; Frank, 2014) assists neurobiologist in identifying multiple psychiatric disorder related genes and their underlying molecular mechanisms (Sun and Xie, 2012; Tian et al., 2017). Knockdown or deactivation (hypomorphic allele) of *Drosophila Tao* increases the number of NMJ (buttons, the single NMJ structure; Politano et al., 2019). However, it seems *Drosophila Tao* that regulates NMJ development is not dependent on the Hippo/MST pathway as it is in hippocampal neurons (Ultanir et al., 2014) but it could negatively regulate BMP signaling as reduction of *Drosophila* Tao leads to an increase in both nucleic pMad levels and BMP target gene expression in motor neuron (Politano et al., 2019). However, another study recently showed that knockdown of *Tao* could decrease the NMJ number (Dulovic-Mahlow et al., 2019). Thus, both studies indicated the important role of *Drosophila Tao* in synaptic development, while the underlying mechanisms for the opposite phenotypes observed independently remains to be illustrated.

We recently developed a novel model for studying synaptic development in *Drosophila larvae* (Tenedini et al., 2019) to compensate for the NMJ model. At larvae stage, peripheral Class IV da (C4da) sensory neurons directly come into contact with a pair of interneurons A08n that can form a functional synaptic structure (Town et al., 2014; Hu et al., 2017; Kaneko et al., 2017), which is closer to mammalian synapses when compared with the classic NMJ synaptic system. By using the Syp-GRASP technique (Macpherson et al., 2015) to label C4da-A08n synapses, we found that loss of Tao results in exuberant postsynaptic (comparable to spine structure) specializations and aberrant connectivity during larval growth. Using functional imaging and a behavioral analysis we showed that loss of *Drosophila* *Tao* could induce ectopic functional synapses formation of the A08n neuron with other types (C3da) of neurons and resulted in altered behavioral responses in a connection-specific manner (Tenedini et al., 2019), indicating that TAO kinase mutations, like other NDDs susceptive genes, can induce abnormal behaviors partially from improper establishment of neural circuits (Kida and Kato, 2015; Kaiser et al., 2017; Südhof, 2017).

### TAO Kinases Control Neuronal Apoptosis and Maturation *via* the JNK Signaling Cascade

The association between neuronal apoptosis and NDDs is not well documented. A previous study showed that overexpression (OE) of the full length or kinase domain of human TAO1 in human neuroblastoma SH-SY5Y cells resulted in cellular apoptosis which was indicated by elevated caspase-3 activity. However, OE of the regulatory domain of TAO1 in SH-SY5Y did not appear to have an obvious change (Wu and Wang, 2008). Importantly, OE of TAO1 induced elevated caspase-3-like activity and apoptosis of neuroblastoma could be reduced by JNK inhibitor SP600125 to some extent. In addition, OE of rat TAO3 in PC12 (a widely used cell line with the properties of intersecting neurons) resulted in elevation of the expression level of BimEL (Wakabayashi et al., 2005), a protein with apoptotic activity. Recently, it was also reported that TAO1 protected MCAO-induced cerebral ischemic stroke by decreasing the pro-inflammatory factors and apoptosis *via* PI3K/AKT and MAPK signaling pathways (Li et al., 2019), which further indicates the critical roles of TAO kinases in neuronal apoptosis. Those preliminary studies suggest an underestimated function of TAO kinases in neuronal apoptosis.

Furthermore, TAO2 may play roles in the regulation of neuronal maturation. A recent study showed that BDNF could regulate cortical GABAergic interneuron maturation in a TAO2-JNK signaling pathway in a 16p11.2 duplication mouse model (Willis et al., 2020). Cultured neurons from 16p11.2 duplication mice exhibit an abnormal interneuron developmental phenotype that may be involved in a premature closure of the critical period which is likely to be driven by OE of TAO2 and the subsequent over-activity of JNK, since pharmacological inhibition of TAO kinase could alleviate the 16p11.2 duplication phenotype. Given the importance of parvalbumin (PBV+) interneurons in the regulation of excitatory/inhibitory balance within cortical regions, accelerated GABAergic development by TAO2 over-activity may lead to dysregulated network activity and synaptic connectivity (Willis et al., 2020). In addition, it was reported that OE of human TAO1 could also prevent maturation of cultured primary hippocampal neurons from mice (Woerden et al., 2021), while the role of JNKs is involvement is yet to be determined.

### TAO Kinases Regulate Neuronal Migration

A recent study investigated a possible function of *TAOK1* in neuronal migration, a process that is critical for normal brain development (Woerden et al., 2021). By employing the *in utero* electroporation in mice at embryonic day 14.5 (a well-established time window when immature neurons generated from progenitor cells start to migrate to their final destination within the cortical plate, which will eventually form the cerebral cortex layer 2/3), the authors found that knockdown of *Taok1* resulted in a clear migration deficit of the neurons when compared to control neurons from postnatal day 1 (p1) to P7, when only 75% of the *Taok1* knockdown neurons were present in cerebral cortex layer 2/3, compared to 95% in control conditions (Woerden et al., 2021). Similar migration deficits were observed when transfection of several NDDs, linked human *TAOK1* variants in developing mouse brains including c.500T>G and c.943C>T (see Table 1), suggesting a possible role of *TAOK1* in neuronal migration during early human brain development.

## Conclusion and Perspective

There is solid evidence showing that all TAO kinase members are involved in NDDs including ASD, schizophrenia, and language/speech development delays, implying an indispensable function of the TAO kinase family in neurodevelopment. Although several animal models from *Drosophila* to knockout mice provides us with a preliminary role of TAO kinases in neuronal development, survival and maturation, the detailed mechanisms of how TAO kinases contribute to NDDs still need to be investigated.

First, the neurobiological functions of TAO kinases should be further investigated. At the molecular level, identification of specific up- and down-stream substrates for TAO kinases in different model systems, especially by combining human induced pluripotent stem cell (iPSC) from patients, is crucial for a better understanding of the mechanisms leading to the onset of a disease-phenotype (Parenti et al., 2020; Zhang et al., 2020). At cellular level, previous reports focus on the cell-autonomous role of TAO kinases in regulating the development of dendrites and dendritic spines. Whether cell-non-autonomous (e.g., different types of glia cells) roles of TAO kinases in neuronal development and functions are present is not reported. In addition, TAO2 was identified to affect axonal development (de Anda et al., 2012). Whether the mechanisms of TAO kinases controlling dendrite/spine development is distinct from axonal development remains unknown. To confirm and further characterize the TAO kinases functions in neurodevelopment or NDDs, non-human primate models are a promising direction.

Second, an obvious question on how Tao kinases contribute to establish and/or modulate disease-related circuits remains unanswered. In *Taok2* knockout mice, the oscillatory events were similar in the PFC and slightly decreased in the HC compared to WT littermates. However, the duration, amplitude, and power in theta (4–7, 12 Hz), beta (12–30 Hz), gamma (30–100 Hz) frequency ranges and coherence within the beta band were significantly increased, suggesting alterations in HC and PFC connectivity (Richter et al., 2018).The limitation of this study is that the animals are studied in an anesthetized status, which may affect the actual physiological responses (Chini et al., 2019). In addition, whether restoring the oscillation pattern by optogenetic stimulation could rescue related phenotypes is also an interesting question that is worth further investigation. A recent study showed a disruption of hippocampal-orbitofrontal-amygdala connectivity in 16p11.2 duplication mice. Whether and how much *Taok2* contributes to this defect needs to be investigated (Bristow et al., 2020).

In addition, the possible non-neurological functions of TAO kinases in developing NDDs should also be highlighted. Increasing evidence suggests a non-neurological role in NDDs including immunity, gut, and microbiota (Cryan and Dinan, 2012; Sharon et al., 2016; Dinan and Cryan, 2017; Stefano et al., 2018; Fattorusso et al., 2019; Pape et al., 2019). Studies investigating the brain-gut axis demonstrate a critical role for the gut microbiota in orchestrating brain development and behavior, and the immune system is emerging as an important regulator of these interactions. Accordingly, both the gut microbiota and immune system are implicated in the etiopathogenesis or manifestation of NDDs (Fung et al., 2017). TAO kinases were reported to regulate immunity (Ormonde et al., 2018, 2019; Zhang et al., 2018), gut development (Huang et al., 2014) and likely in maintenance of microbiota integration since MAPKs are involved (Thomas and Versalovic, 2010). Thus, the possible non-neurological role of TAO kinases in NDDs should be seriously considered. TAO kinases are ubiquitously expressed in all tissues including in the immune and gastrointestinal system (Duan et al., 2020). However, it is still unknown whether TAO kinases play any role in the regulation of the immune system and the coordination of brain-gut axis functions that affects neurodevelopment and contributes to NDDs.

**Figure 4:**
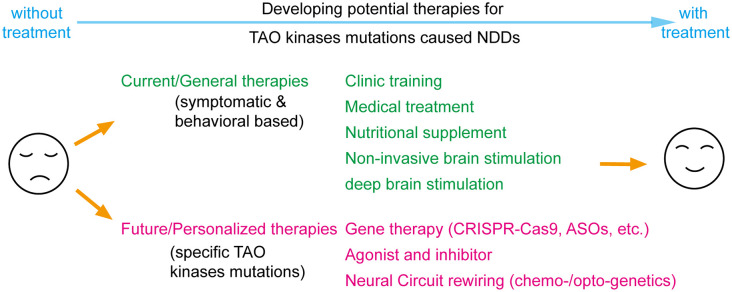
Perspectives on developing potential therapies for TAO kinases mutations caused neurodevelopmental disorders (NDDs). Current therapies include most symptomatic and behavioral based treatments which could be applied to all factors caused NDDs including traditional clinical training, medical treatment, nutritional supplementation, and brain stimulations. With significant progress in biotechnology, the concept for future therapies could focus on personalized therapy by specifically targeting TAO kinase mutations by gene therapy, developing specific agonist or inhibitors and rewiring neural circuits with chemo- or optogenetics.

The eventual goal of investigating the roles and functions of TAO kinases in NDDs is to develop potential therapeutic approaches (Figure 4). Although several above-mentioned basic research discoveries may provide directions for developing potential therapies, clinical trails or even pre-clinical studies on treatment of TAO kinases related NDDs are still lacking. Current clinical training intervention and medical treatments offered for NDDs are symptomatic and behavioral based therapy (Levy and Barak, 2021), which could be applied to any possible factors caused by NDDs. In addition, nutritional supplements (Chang and Su, 2020), non-invasive brain stimulation [including transcranial direct current stimulation (tDCS) and transcranial magnetic stimulation (TMS)] (Khaleghi et al., 2020), and deep brain stimulation in certain cases (Beszłej et al., 2019; Lin et al., 2019) are being or will be applied in clinical treatments of NDDs.

Although those general treatments could rescue certain symptoms and behaviors, developing precision medicine that directly and specifically targets disease-causing mutations would be more beneficial (Javed et al., 2020). To functionally rescue TAO kinases mutations caused NDDs, the principle of a long-term rebalancing of their kinase activity should always be followed. One possible strategy is to develop agonists and inhibitors with high specificity and potency for TAO kinases (Fang et al., 2020; Ye et al., 2020). Similar to all standardized drug development processes, the efficacy and toxicity in animal models should be cautiously evaluated before these candidates are applied in human clinical trials. In addition, with the significant progress of gene editing and manipulation in the last decade, gene therapy techniques are a promising direction for personalized medical treatment including CRISPR-Cas9 gene-editing for mutation replacement and antisense oligonucleotides (ASOs) for modifying protein levels (Levy and Barak, 2021). It should be noted that most of those gene therapies are still in preclinical testing stages and the number of successful clinical trials is quite limited because it is faced with critical issues when implemented in *in vivo*, such as limited efficiency, off-target effects, time window and delivery methods, all suggesting that much more effort is required to improve the bench-to-bedside success rate (Levy and Barak, 2021). Theoretically, all those strategies could also be applied to the specific down-stream targets (like Septin7, myosin Va, JNK and RhoA) of TAO kinases which requires further exploration of the signaling network of TAO kinases.

Alternatively, another option is bypassing molecular deficits and directly treating the neural circuit level deficits (Javed et al., 2020). It is widely accepted that NDDs arise from the alteration of normal brain developmental trajectories disrupting the function of specific neural circuits caused by genetic mutations (del Pino et al., 2018). To rewire the neural circuits by chemo- and optogenetic manipulations is a promising strategy to treat NDDs (Ferguson and Gao, 2018). The successful application of optogenetic tools to rebuild the functional neural circuit from dorsal raphe to nucleus accumbens that rescues the social defects in 16p11.2 del mice (Walsh et al., 2018) may prove therapeutically beneficial. Thus, the specific neural circuit defects in TAO kinases related NDDs requires further exploration.

In short, human genetic studies and animal models have linked all TAO kinase members to NDDs such as ASD and schizophrenia. The underlying mechanisms of how TAO kinases regulate neurodevelopment, and how their mutations contribute to NDDs are only just emerging. To dissect more detailed and precise mechanisms is a prerequisite in developing personalized therapies for TAO kinases mutations caused NDDs.

## Author Contributions

CH wrote the manuscript, prepared the figures, and finalized the manuscript. LX contributed to writing and commenting. PF and QY contributed to figure and table preparation. All authors contributed to the article and approved the submitted version.

## Conflict of Interest

The authors declare that the research was conducted in the absence of any commercial or financial relationships that could be construed as a potential conflict of interest.

## Footnotes

^1^https://www.gtexportal.org/

## References

[B1] Abdellaoui A. Sanchez-Roige S. Sealock J. Treur J. L. Dennis J. Fontanillas P.. (2019). Phenome-wide investigation of health outcomes associated with genetic predisposition to loneliness. Hum. Mol. Genet. 28, 3853–3865. 10.1093/hmg/ddz219 31518406 PMC6935385

[B2] Baselmans B. M. L. Jansen R. Ip H. F. van Dongen J. Abdellaoui A. van de Weijer M. P.. (2019). Multivariate genome-wide analyses of the well-being spectrum. Nat. Genet. 51, 445–451. 10.1038/s41588-018-0320-8 30643256

[B3] Batool S. Raza H. Zaidi J. Riaz S. Hasan S. Syed N. I. (2019). Synapse formation: from cellular and molecular mechanisms to neurodevelopmental and neurodegenerative disorders. J. Neurophysiol. 121, 1381–1397. 10.1152/jn.00833.2018 30759043

[B4] Benson D. L. Colman D. R. Huntley G. W. (2001). Molecules, maps and synapse specificity. Nat. Rev. Neurosci. 2, 899–909. 10.1038/35104078 11733797

[B5] Berman K. S. Hutchison M. Avery L. Cobb M. H. (2001). kin-18, a *C. elegans* protein kinase involved in feeding. Gene 279, 137–147. 10.1016/s0378-1119(01)00752-1 11733138 PMC4441751

[B6] Beszłej J. A. Wieczorek T. Kobyłko A. Piotrowski P. Siwicki D. Weiser A.. (2019). Deep brain stimulation: new possibilities for the treatment of mental disorders. Psychiatr. Pol. 53, 789–806. 10.12740/PP/OnlineFirst/103090 31760410

[B7] Boggiano J. C. Vanderzalm P. J. Fehon R. G. (2011). Tao-1 phosphorylates Hippo/MST kinases to regulate the Hippo-Salvador-Warts tumor suppressor pathway. Dev. Cell 21, 888–895. 10.1016/j.devcel.2011.08.028 22075147 PMC3217187

[B8] Bowen J. R. Hwang D. Bai X. Roy D. Spiliotis E. T. (2011). Septin GTPases spatially guide microtubule organization and plus end dynamics in polarizing epithelia. J. Cell Biol. 194, 187–197. 10.1083/jcb.201102076 21788367 PMC3144415

[B9] Bristow G. C. Thomson D. M. Openshaw R. L. Mitchell E. J. Pratt J. A. Dawson N.. (2020). 16p11 duplication disrupts hippocampal-orbitofrontal-amygdala connectivity, revealing a neural circuit endophenotype for schizophrenia. Cell Rep. 31:107536. 10.1016/j.celrep.2020.107536 32320645

[B10] Broadie K. Bate M. (1995). The *Drosophila* NMJ: a genetic model system for synapse formation and function. Semin. Dev. Biol. 6, 221–231. 10.1016/S1044-5781(06)80031-9

[B11] Cargnello M. Roux P. P. (2011). Activation and function of the MAPKs and their substrates, the MAPK-activated protein kinases. Microbiol. Mol. Biol. Rev. 75, 50–83. 10.1128/MMBR.00031-10 21372320 PMC3063353

[B12] Chang J. P. C. Su K. P. (2020). Nutritional neuroscience as mainstream of psychiatry: the evidence-based treatment guidelines for using omega-3 fatty acids as a new treatment for psychiatric disorders in children and adolescents. Clin. Psychopharmacol. Neurosci. 18, 469–483. 10.9758/cpn.2020.18.4.469 33124582 PMC7609218

[B13] Chen Z. Hutchison M. Cobb M. H. (1999). Isolation of the protein kinase TAO2 and identification of its mitogen-activated protein kinase/extracellular signal-regulated kinase kinase binding domain. J. Biol. Chem. 274, 28803–28807. 10.1074/jbc.274.40.28803 10497253

[B14] Chini M. Gretenkord S. Kostka J. K. Pöpplau J. A. Cornelissen L. Berde C. B.. (2019). Neural correlates of anesthesia in newborn mice and humans. Front. Neural Circuits 13, 1–13. 10.3389/fncir.2019.00038 31191258 PMC6538977

[B15] Chung H. L. Augustine G. J. Choi K. W. (2016). *Drosophila* schip1 links expanded and tao-1 to regulate hippo signaling. Dev. Cell 36, 511–524. 10.1016/j.devcel.2016.02.004 26954546

[B16] Coe B. P. Stessman H. A. F. Sulovari A. Geisheker M. R. Bakken T. E. Lake A. M.. (2019). Neurodevelopmental disease genes implicated by de novo mutation and copy number variation morbidity. Nat. Genet. 51, 106–116. 10.1038/s41588-018-0288-4 30559488 PMC6309590

[B17] Coll-Tane M. Krebbers A. Castells-Nobau A. Zweier C. Schenck A. (2019). Intellectual disability and autism spectrum disorders “on the fly”: insights from *Drosophila*. Dis. Models. Mech. 12:dmm039180. 10.1242/dmm.039180 31088981 PMC6550041

[B18] Colón-Ramos D. A. (2009). Chapter 2-synapse formation in developing neural circuits. Curr. Top. Dev. Biol. 87, 53–79. 10.1016/S0070-2153(09)01202-2 19427516 PMC7649972

[B19] Conde C. Cáceres A. (2009). Microtubule assembly, organization and dynamics in axons and dendrites. Nat. Rev. Neurosci. 10, 319–332. 10.1038/nrn2631 19377501

[B20] Cook-Sather S. D. Li J. Goebel T. K. Sussman E. M. Rehman M. A. Hakonarson H. (2014). TAOK3, a novel genome-wide association study locus associated with morphine requirement and postoperative pain in a retrospective pediatric day surgery population. Pain 155, 1773–1783. 10.1016/j.pain.2014.05.032 24909733 PMC4157963

[B21] Cooper G. M. Coe B. P. Girirajan S. Rosenfeld J. A. Vu T. H. Baker C.. (2011). A copy number variation morbidity map of developmental delay. Nat. Genet. 43, 838–846. 10.1038/ng.909 21841781 PMC3171215

[B22] Copf T. (2015). Importance of gene dosage in controlling dendritic arbor formation during development. Eur. J. Neurosci. 42, 2234–2249. 10.1111/ejn.13002 26108333

[B23] Cryan J. F. Dinan T. G. (2012). Mind-altering microorganisms: the impact of the gut microbiota on brain and behaviour. Nat. Rev. Neurosci. 13, 701–712. 10.1038/nrn3346 22968153

[B24] Dan I. Watanabe N. M. Kusumi A. (2001). The Ste20 group kinases as regulators of MAP kinase cascades. Trends Cell Biol. 11, 220–230. 10.1016/s0962-8924(01)01980-8 11316611

[B25] de Anda F. C. Rosario A. L. Durak O. Tran T. Gräff J. Meletis K.. (2012). Autism spectrum disorder susceptibility gene TAOK2 affects basal dendrite formation in the neocortex. Nat. Neurosci. 15, 1022–1031. 10.1038/nn.3141 22683681 PMC4017029

[B26] Deciphering Developmental Disorders Study (2017). Prevalence and architecture of *de novo* mutations in developmental disorders. Nature 542, 433–438. 10.1038/nature21062 28135719 PMC6016744

[B27] del Pino I. Rico B. Marín O. (2018). Neural circuit dysfunction in mouse models of neurodevelopmental disorders. Curr. Opin. Neurobiol. 48, 174–182. 10.1016/j.conb.2017.12.013 29329089

[B28] Deng J. Erdjument-Bromage H. Neubert T. A. (2019). Quantitative comparison of proteomes using SILAC. Curr. Protoc. Protein Sci. 95, 1–14. 10.1002/cpps.74 30238645 PMC6342620

[B29] Dinan T. G. Cryan J. F. (2017). Brain-gut-microbiota axis and mental health. Psychosom. Med. 79, 920–926. 10.1097/PSY.0000000000000519 28806201

[B30] Drewes G. Ebneth A. Preuss U. Mandelkow E. M. Mandelkow E. (1997). MARK, a novel family of protein kinases that phosphorylate microtubule- associated proteins and trigger microtubule disruption. Cell 89, 297–308. 10.1016/s0092-8674(00)80208-1 9108484

[B31] Duan Q. Ye J. Shi M. Chen W. Zhu F. (2020). Research advances in the molecular functions and relevant diseases of TAOKs, novel STE20 kinase family members. Curr. Pharm. Des. 26, 1–12. 10.2174/1381612826666200203115458 32013821

[B32] Dulovic-Mahlow M. Trinh J. Kandaswamy K. K. Braathen G. J. Di Donato N. Rahikkala E.. (2019). *De novo* variants in TAOK1 cause neurodevelopmental disorders. Am. J. Hum. Genet. 105, 213–220. 10.1016/j.ajhg.2019.05.005 31230721 PMC6612514

[B34] Fang C. Y. Lai T. C. Hsiao M. Chang Y. C. (2020). The diverse roles of tao kinases in health and diseases. Int. J. Mol. Sci. 21, 1–21. 10.3390/ijms21207463 33050415 PMC7589832

[B35] Fattorusso A. Di Genova L. Dell’isola G. B. Mencaroni E. Esposito S. (2019). Autism spectrum disorders and the gut microbiota. Nutrients 11:521. 10.3390/nu11030521 30823414 PMC6471505

[B36] Ferguson B. R. Gao W. J. (2018). Pv interneurons: critical regulators of E/I balance for prefrontal cortex-dependent behavior and psychiatric disorders. Front. Neural Circuits 12, 1–13. 10.3389/fncir.2018.00037 29867371 PMC5964203

[B37] Firth H. V. Wright C. F. (2011). The deciphering developmental disorders (DDD) study. Dev. Med. Child Neurol. 53, 702–703. 10.1111/j.1469-8749.2011.04032.x 21679367

[B38] Fischbach G. D. Lord C. (2010). The simons simplex collection: a resource for identification of autism genetic risk factors. Neuron 68, 192–195. 10.1016/j.neuron.2010.10.006 20955926

[B39] Fitzgerald T. W. Gerety S. S. Jones W. D. Van Kogelenberg M. King D. A. McRae J.. (2015). Large-scale discovery of novel genetic causes of developmental disorders. Nature 519, 223–228. 10.1038/nature14135 25533962 PMC5955210

[B40] Frank C. A. (2014). Homeostatic plasticity at the *Drosophila* neuromuscular junction. Neuropharmacology 78, 63–74. 10.1016/j.neuropharm.2013.06.015 23806804 PMC3830618

[B41] Freitag C. M. Luders E. Hulst H. E. Narr K. L. Thompson P. M. Toga A. W.. (2009). Total brain volume and corpus callosum size in medication-naïve adolescents and young adults with autism spectrum disorder. Biol. Psychiatry 66, 316–319. 10.1016/j.biopsych.2009.03.011 19409535 PMC3299337

[B42] Fung T. C. Olson C. A. Hsiao E. Y. (2017). Interactions between the microbiota, immune and nervous systems in health and disease. Nat. Neurosci. 20, 145–155. 10.1038/nn.4476 28092661 PMC6960010

[B43] Gatto C. L. Broadie K. (2011). *Drosophila* modeling of heritable neurodevelopmental disorders. Curr. Opin. Neurobiol. 21, 834–841. 10.1016/j.conb.2011.04.009 21596554 PMC3172335

[B44] Giacomini C. Koo C.-Y. Yankova N. Tavares I. A. Wray S. Noble W.. (2018). A new TAO kinase inhibitor reduces tau phosphorylation at sites associated with neurodegeneration in human tauopathies. Acta Neuropathol. Commun. 6:37. 10.1186/s40478-018-0539-8 29730992 PMC5937037

[B45] Gilman S. R. Chang J. Xu B. Bawa T. S. Gogos J. A. Karayiorgou M.. (2012). Diverse types of genetic variation converge on functional gene networks involved in schizophrenia. Nat. Neurosci. 15, 1723–1728. 10.1038/nn.3261 23143521 PMC3689007

[B46] Grueber W. B. Jan L. Y. Jan Y. N. (2002). Tiling of the *Drosophila* epidermis by multidendritic sensory neurons. Development 129, 2867–2878. 12050135 10.1242/dev.129.12.2867

[B47] Guhathakurta P. Prochniewicz E. Thomas D. D. (2018). Actin-myosin interaction: Structure, function and drug discovery. Int. J. Mol. Sci. 19:2628. 10.3390/ijms19092628 30189615 PMC6163256

[B48] Gutteridge T. Kumaran M. Ghosh S. Fainsinger R. Klepstad P. Tarumi Y.. (2018). Single-nucleotide polymorphisms in TAOK3 are associated with high opioid requirement for pain management in patients with advanced cancer admitted to a tertiary palliative care unit. J. Pain Symptom Manage. 56, 560–566. 10.1016/j.jpainsymman.2018.07.011 30031856

[B49] Hanly C. Shah H. Au P. Y. B. Murias K. (2021). Description of neurodevelopmental phenotypes associated with 10 genetic neurodevelopmental disorders: a scoping review. Clin. Genet. 99, 335–346. 10.1111/cge.13882 33179249

[B50] Hardan A. Y. Libove R. A. Keshavan M. S. Melhem N. M. Minshew N. J. (2009). A preliminary longitudinal magnetic resonance imaging study of brain volume and cortical thickness in autism. Biol. Psychiatry 66, 320–326. 10.1016/j.biopsych.2009.04.024 19520362 PMC2905654

[B111] Harrington W. F. Rodgers M. E. (1984). MYOSIN. Annu. Rev. Biochem. 53, 35–73. 10.1146/annurev.bi.53.070184.000343 6383197

[B51] Harvey K. F. Pfleger C. M. Hariharan I. K. (2003). The *Drosophila* Mst ortholog, hippo, restricts growth and cell proliferation and promotes apoptosis. Cell 114, 457–467. 10.1016/s0092-8674(03)00557-9 12941274

[B52] Hazlett H. C. Poe M. Gerig G. Smith R. G. Provenzale J. Ross A.. (2005). Magnetic resonance imaging and head circumference study of brain size in autism: birth through age 2 years. Arch. Gen. Psychiatry 62, 1366–1376. 10.1001/archpsyc.62.12.1366 16330725

[B53] Hoogenraad C. C. Bradke F. (2009). Control of neuronal polarity and plasticity—a renaissance for microtubules? Trends Cell Biol. 19, 669–676. 10.1016/j.tcb.2009.08.006 19801190

[B54] Hu C. Kanellopoulos A. K. Richter M. Petersen M. Konietzny A. Tenedini F. M.. (2020). Conserved tao kinase activity regulates dendritic arborization, cytoskeletal dynamics and sensory function in *Drosophila*. J. Neurosci. 40, 1819–1833. 10.1523/JNEUROSCI.1846-19.2020 31964717 PMC7046460

[B55] Hu C. Petersen M. Hoyer N. Spitzweck B. Tenedini F. Wang D.. (2017). Sensory integration and neuromodulatory feedback facilitate *Drosophila* mechanonociceptive behavior. Nat. Neurosci. 20, 1085–1095. 10.1038/nn.4580 28604684 PMC5931224

[B56] Hu J. Bai X. Bowen J. R. Dolat L. Korobova F. Yu W.. (2012). Septin-driven coordination of actin and microtubule remodeling regulates the collateral branching of axons. Curr. Biol. 22, 1109–1115. 10.1016/j.cub.2012.04.019 22608511 PMC3381998

[B57] Huang X. Shi L. Cao J. He F. Li R. Zhang Y.. (2014). The Sterile 20-Like kinase tao controls tissue homeostasis by regulating the hippo pathway in drosophila adult midgut. J. Genet. Genomics 41, 429–438. 10.1016/j.jgg.2014.05.007 25160975

[B58] Hutchison M. Berman K. S. Cobb M. H. (1998). Isolation of TAO1, a protein kinase that activates MEKs in stress- activated protein kinase cascades. J. Biol. Chem. 273, 28625–28632. 10.1074/jbc.273.44.28625 9786855

[B59] Im S. H. Galko M. J. (2012). Pokes, sunburn and hot sauce: *Drosophila* as an emerging model for the biology of nociception. Dev. Dyn. 241, 16–26. 10.1002/dvdy.22737 21932321 PMC3258975

[B60] Iossifov I. O’Roak B. J. Sanders S. J. Ronemus M. Krumm N. Levy D.. (2014). The contribution of *de novo* coding mutations to autism spectrum disorder. Nature 515, 216–221. 10.1038/nature13908 25363768 PMC4313871

[B61] Jan Y. N. Jan L. Y. (2001). Dendrites. Genes Dev. 15, 2627–2641. 10.1101/gad.916501 11641269

[B62] Jan Y.-N. Jan L. Y. (2010). Branching out: mechanisms of dendritic arborization. Nat. Rev. Neurosci. 11, 316–328. 10.1038/nrn2836 20404840 PMC3079328

[B63] Javed S. Selliah T. Lee Y. J. Huang W. H. (2020). Dosage-sensitive genes in autism spectrum disorders: From neurobiology to therapy. Neurosci. Biobehav. Rev. 118, 538–567. 10.1016/j.neubiorev.2020.08.009 32858083

[B64] Johnson G. L. Lapadat R. (2002). Mitogen-activated protein kinase pathways mediated by ERK, JNK and p38 protein kinases. Science 298, 1911–1912. 10.1126/science.1072682 12471242

[B65] Kaiser T. Zhou Y. Feng G. (2017). Animal models for neuropsychiatric disorders: prospects for circuit intervention. Curr. Opin. Neurobiol. 45, 59–65. 10.1016/j.conb.2017.03.010 28419975

[B66] Kaneko T. Macara A. M. Li R. Hu Y. Iwasaki K. Dunnings Z.. (2017). Correction: serotonergic modulation enables pathway-specific plasticity in a developing sensory circuit in drosophila. Neuron 95:722. 10.1016/j.neuron.2017.06.034 28772126

[B67] Kapfhamer D. King I. Zou M. E. Lim J. P. Heberlein U. Wolf F. W. (2012). JNK pathway activation is controlled by Tao/TAOK3 to modulate ethanol sensitivity. PLoS One 7:e50594. 10.1371/journal.pone.0050594 23227189 PMC3515618

[B68] Kapfhamer D. Taylor S. Zou M. E. Lim J. P. Kharazia V. Heberlein U. (2013). Taok2 controls behavioral response to ethanol in mice. Genes Brain Behav. 12, 87–97. 10.1111/j.1601-183X.2012.00834.x 22883308 PMC4011144

[B69] Kapitein L. C. Hoogenraad C. C. (2015). building the neuronal microtubule cytoskeleton. Neuron 87, 492–506. 10.1016/j.neuron.2015.05.046 26247859

[B70] Kapitein L. C. van Bergeijk P. Lipka J. Keijzer N. Wulf P. S. Katrukha E. A.. (2013). Myosin-V opposes microtubule-based cargo transport and drives directional motility on cortical actin. Curr. Biol. 23, 828–834. 10.1016/j.cub.2013.03.068 23602478

[B71] Khaleghi A. Zarafshan H. Vand S. R. Mohammadi M. R. (2020). Effects of non-invasive neurostimulation on autism spectrum disorder: a systematic review. Clin. Psychopharmacol. Neurosci. 18, 527–552. 10.9758/cpn.2020.18.4.527 33124586 PMC7609207

[B72] Kida S. Kato T. (2015). Microendophenotypes of psychiatric disorders: phenotypes of psychiatric disorders at the level of molecular dynamics, synapses, neurons and neural circuits. Curr. Mol. Med. 15, 111–118. 10.2174/1566524015666150303002128 25732153 PMC4460283

[B73] King I. Heberlein U. (2011). Tao kinases as coordinators of actin and microtubule dynamics in developing neurons. Commun. Integr. Biol. 4, 554–556. 10.4161/cib.4.5.16051 22046460 PMC3204126

[B74] Komis G. Illés P. Beck M. Šamaj J. (2011). Microtubules and mitogen-activated protein kinase signalling. Curr. Opin. Plant Biol. 14, 650–657. 10.1016/j.pbi.2011.07.008 21839668

[B75] Konietzny A. Bär J. Mikhaylova M. (2017). Dendritic actin cytoskeleton: structure, functions and regulations. Front. Cell. Neurosci. 11:147. 10.3389/fncel.2017.00147 28572759 PMC5435805

[B76] Krishnan A. Zhang R. Yao V. Theesfeld C. L. Wong A. K. Tadych A.. (2016). Genome-wide prediction and functional characterization of the genetic basis of autism spectrum disorder. Nat. Neurosci. 19, 1454–1462. 10.1038/nn.4353 27479844 PMC5803797

[B77] Krystal J. H. State M. W. (2014). Psychiatric disorders: diagnosis to therapy. Cell 157, 201–214. 10.1016/j.cell.2014.02.042 24679536 PMC4104191

[B78] Kwan C. Gitimoghaddam M. Collet J. P. (2020). Effects of social isolation and loneliness in children with neurodevelopmental disabilities: a scoping review. Brain Sci. 10, 1–36. 10.3390/brainsci10110786 33126519 PMC7693393

[B79] Kyosseva S. V. (2004). Mitogen-activated protein kinase signaling. Int. Rev. Neurobiol. 59, 201–220. 10.1016/S0074-7742(04)59008-6 15006489

[B80] Lai M. C. Lombardo M. V. Baron-Cohen S. (2014). Autism. Lancet 383, 896–910. 10.1016/S0140-6736(13)61539-1 24074734

[B81] Levy G. Barak B. (2021). Postnatal therapeutic approaches in genetic neurodevelopmental disorders. Neural Regen. Res. 16, 414–422. 10.4103/1673-5374.293133 32985459 PMC7996025

[B82] Li J. Liu Z. Wang L. Xu H. Wang Y. (2019). Thousand one kinase 1 protects MCAO-induced cerebral ischemic stroke in rats by decreasing apoptosis and pro-inflammatory factors. Biosci. Rep. 39, 1–11. 10.1042/BSR20190749 31652447 PMC6822489

[B83] Lin T. C. Lo Y. C. Lin H. C. Li S. J. Lin S. H. Wu H. F.. (2019). MR imaging central thalamic deep brain stimulation restored autistic-like social deficits in the rat. Brain Stimul. 12, 1410–1420. 10.1016/j.brs.2019.07.004 31324604

[B84] Liu T. Rohn J. L. Picone R. Kunda P. Baum B. (2010). Tao-1 is a negative regulator of microtubule plus-end growth. J. Cell Sci. 123, 2708–2716. 10.1242/jcs.068726 20647372 PMC2915876

[B85] Lombardo A. T. Nelson S. R. Kennedy G. G. Trybus K. M. Walcott S. Warshaw D. M. (2019). Myosin Va transport of liposomes in three-dimensional actin networks is modulated by actin filament density, position and polarity. Proc. Natl. Acad. Sci. U S A 116, 8326–8335. 10.1073/pnas.1901176116 30967504 PMC6486769

[B86] Macpherson L. J. Zaharieva E. E. Kearney P. J. Alpert M. H. Lin T. Y. Turan Z.. (2015). Dynamic labeling of neural connections in multiple colours by trans-synaptic fluorescence complementation. Nat. Commun. 6:10024. 10.1038/ncomms10024 26635273 PMC4686661

[B87] Malhotra D. McCarthy S. Michaelson J. J. Vacic V. Burdick K. E. Yoon S.. (2011). High frequencies of *de novo* cnvs in bipolar disorder and schizophrenia. Neuron 72, 951–963. 10.1016/j.neuron.2011.11.007 22196331 PMC3921625

[B300] Masters T. A. Kendrick-Jones J. Buss F. (2016). “Myosins: domain organisation, motor properties, physiological roles and cellular functions,” in The Actin Cytoskeleton. Handbook of Experimental Pharmacology, vol 235, ed Jockusch B. (Cham: Springer), 77–122. 10.1007/164_2016_29 27757761

[B88] Mavrakis M. Azou-Gros Y. Tsai F. C. Alvarado J. Bertin A. Iv F.. (2014). Septins promote F-actin ring formation by crosslinking actin filaments into curved bundles. Nat. Cell Biol. 16, 322–334. 10.1038/ncb2921 24633326

[B89] McAllister A. K. (2007). Dynamic aspects of cns synapse formation. Annu. Rev. Neurosci. 30, 425–450. 10.1146/annurev.neuro.29.051605.112830 17417940 PMC3251656

[B90] McCarthy S. E. Makarov V. Kirov G. Addington A. M. McClellan J. Yoon S.. (2009). Microduplications of 16p11.2 are associated with schizophrenia. Nat. Genet. 41, 1223–1227. 10.1038/ng.474 19855392 PMC2951180

[B91] Menon K. P. Carrillo R. A. Zinn K. (2013). Development and plasticity of the *Drosophila* larval neuromuscular junction. Wiley Interdiscip. Rev. Dev. Biol. 2, 647–670. 10.1002/wdev.108 24014452 PMC3767937

[B92] Miller C. J. Lou H. J. Simpson C. Van De Kooij B. Hak Ha B. Fisher O. S.. (2019). Comprehensive profiling of the STE20 kinase family defines features essential for selective substrate targeting and signaling output. PLoS One 17:e2006540. 10.1371/journal.pbio.2006540 30897078 PMC6445471

[B93] Mitsopoulos C. Zihni C. Garg R. Ridley A. J. Morris J. D. H. (2003). The prostate-derived sterile 20-like kinase (PSK) regulates microtubule organization and stability. J. Biol. Chem. 278, 18085–18091. 10.1074/jbc.M213064200 12639963

[B94] Moore T. M. Garg R. Johnson C. Coptcoat M. J. Ridley A. J. Morris J. D. (2000). PSK, a novel STE20-like kinase derived from prostatic carcinoma that activates the c-Jun N-terminal kinase mitogen-activated protein kinase pathway and regulates actin cytoskeletal organization. J. Biol. Chem. 275, 4311–4322. 10.1074/jbc.275.6.4311 10660600

[B95] Moyses-Oliveira M. Yadav R. Erdin S. Talkowski M. E. (2020). New gene discoveries highlight functional convergence in autism and related neurodevelopmental disorders. Curr. Opin. Genet. Dev. 65, 195–206. 10.1016/j.gde.2020.07.001 32846283

[B96] Neubauer K. Zieger B. (2017). The mammalian septin interactome. Front. Cell Dev. Biol. 5, 1–9. 10.3389/fcell.2017.00003 28224124 PMC5293755

[B97] Orefice L. L. Mosko J. R. Morency D. T. Wells M. F. Tasnim A. Mozeika S. M.. (2019). Targeting peripheral somatosensory neurons to improve tactile-related phenotypes in ASD models. Cell 178, 867–886.e24. 10.1016/j.cell.2019.07.024 31398341 PMC6704376

[B98] Orefice L. L. Zimmerman A. L. Chirila A. M. Sleboda S. J. Head J. P. Ginty D. D. (2016). Peripheral mechanosensory neuron dysfunction underlies tactile and behavioral deficits in mouse models of ASDs. Cell 166, 299–313. 10.1016/j.cell.2016.05.033 27293187 PMC5567792

[B99] Ormonde J. V. S. Li Z. Stegen C. Madrenas J. (2018). TAOK3 regulates canonical TCR signaling by preventing early SHP-1-mediated inactivation of LCK. J. Immunol. 201, 3431–3442. 10.4049/jimmunol.1800284 30373850

[B100] Ormonde J. V. S. Nie Y. Madrenas J. (2019). TAOK3, a regulator of LCK-SHP-1 crosstalk during TCR signaling. Crit. Rev. Immunol. 39, 59–81. 10.1615/CritRevImmunol.2019030480 31679194

[B101] Papagavriel K. Jones R. Sheehan R. Hassiotis A. Ali A. (2020). The association between loneliness and common mental disorders in adults with borderline intellectual impairment. J. Affect. Disord. 277, 954–961. 10.1016/j.jad.2020.09.005 33065838

[B102] Pape K. Tamouza R. Leboyer M. Zipp F. (2019). Immunoneuropsychiatry—novel perspectives on brain disorders. Nat. Rev. Neurol. 15, 317–328. 10.1038/s41582-019-0174-4 30988501

[B103] Parenti I. Rabaneda L. G. Schoen H. Novarino G. (2020). Neurodevelopmental disorders: from genetics to functional pathways. Trends Neurosci. 43, 608–621. 10.1016/j.tins.2020.05.004 32507511

[B104] Pflanz R. Voigt A. Yakulov T. Jäckle H. (2015). *Drosophila* gene tao-1 encodes proteins with and without a Ste20 kinase domain that affect cytoskeletal architecture and cell migration differently. Open Biol. 5:140161. 10.1098/rsob.140161 25589578 PMC4313371

[B105] Politano S. F. Salemme R. R. Ashley J. López-Rivera J. A. Bakula T. A. Puhalla K. A.. (2019). Tao negatively regulates BMP signaling during neuromuscular junction development in drosophila. Dev. Neurobiol. 79, 335–349. 10.1002/dneu.22681 31002474 PMC6848449

[B106] Poon C. L. C. Lin J. I. Zhang X. Harvey K. F. (2011). The sterile 20-like kinase Tao-1 controls tissue growth by regulating the salvador-warts-hippo pathway. Dev. Cell 21, 896–906. 10.1016/j.devcel.2011.09.012 22075148

[B107] Poon C. L. C. Liu W. Song Y. Gomez M. Kulaberoglu Y. Zhang X.. (2018). A hippo-like signaling pathway controls tracheal morphogenesis in *Drosophila melanogaster*. Dev. Cell 47, 564–575. 10.1016/j.devcel.2018.09.024 30458981 PMC6281297

[B108] Rein B. Yan Z. (2020). 16p11.2 copy number variations and neurodevelopmental disorders. Trends Neurosci. 43, 886–901. 10.1016/j.tins.2020.09.001 32993859 PMC7606557

[B109] Richter M. Murtaza N. Scharrenberg R. White S. H. Johanns O. Walker S.. (2018). Altered TAOK2 activity causes autism-related neurodevelopmental and cognitive abnormalities through RhoA signaling. Mol. Psychiatry 24, 1329–1350. 10.1038/s41380-018-0025-5 29467497 PMC6756231

[B110] Robertson C. E. Baron-Cohen S. (2017). Sensory perception in autism. Nat. Rev. Neurosci. 18, 671–684. 10.1038/nrn.2017.112 28951611

[B112] Sacco R. Gabriele S. Persico A. M. (2015). Head circumference and brain size in autism spectrum disorder: a systematic review and meta-analysis. Psychiatry Res. 234, 239–251. 10.1016/j.pscychresns.2015.08.016 26456415

[B113] Šamaj J. Baluška F. Hirt H. (2004). From signal to cell polarity: mitogen-activated protein kinases as sensors and effectors of cytoskeleton dynamicity. J. Exp. Bot. 55, 189–198. 10.1093/jxb/erh012 14673033

[B114] Sanders S. J. Neale B. M. Huang H. Werling D. M. An J. Y. Dong S.. (2018). Publisher correction: whole genome sequencing in psychiatric disorders: the WGSPD consortium. Nat. Neurosci. 21:1017. 10.1038/s41593-018-0102-8 29549319

[B115] Satterstrom F. K. Kosmicki J. A. Wang J. Breen M. S. De Rubeis S. An J. Y.. (2020). Large-scale exome sequencing study implicates both developmental and functional changes in the neurobiology of autism. Cell 180, 568–584. 10.1016/j.cell.2019.12.036 31981491 PMC7250485

[B116] Sharon G. Sampson T. R. Geschwind D. H. Mazmanian S. K. (2016). The central nervous system and the gut microbiome. Cell 167, 915–932. 10.1016/j.cell.2016.10.027 27814521 PMC5127403

[B117] Shimono K. Fujimoto A. Tsuyama T. Yamamoto-Kochi M. Sato M. Hattori Y.. (2009). Multidendritic sensory neurons in the adult Drosophila abdomen: origins, dendritic morphology and segment- and age-dependent programmed cell death. Neural Dev. 4:37. 10.1186/1749-8104-4-37 19799768 PMC2762467

[B118] Sobreira N. Valle D. Hamosh A. (2015). GeneMatcher: a matching tool for connecting investigators with an interest in the same gene. Hum. Mutat. 36, 928–930. 10.1002/humu.22844 26220891 PMC4833888

[B119] Spiga F. M. Prouteau M. Gotta M. (2013). The TAO kinase KIN-18 regulates contractility and establishment of polarity in the *C. elegans* embryo. Dev. Biol. 373, 26–38. 10.1016/j.ydbio.2012.10.001 23064028

[B120] Stefano G. B. Pilonis N. Ptacek R. Raboch J. Vnukova M. Kream R. M. (2018). Gut, microbiome and brain regulatory axis: relevance to neurodegenerative and psychiatric disorders. Cell. Mol. Neurobiol. 38, 1197–1206. 10.1007/s10571-018-0589-2 29802603 PMC6061125

[B121] Steinman K. J. Spence S. J. Ramocki M. B. Proud M. B. Kessler S. K. Marco E. J.. (2016). 16p11.2 deletion and duplication: characterizing neurologic phenotypes in a large clinically ascertained cohort. Am. J. Med. Genet. Part A 170, 2943–2955. 10.1002/ajmg.a.37820 27410714

[B122] Südhof T. C. (2017). Synaptic neurexin complexes: a molecular code for the logic of neural circuits. Cell 171, 745–769. 10.1016/j.cell.2017.10.024 29100073 PMC5694349

[B123] Sullivan P. F. Geschwind D. H. (2019). Defining the genetic, genomic, cellular and diagnostic architectures of psychiatric disorders. Cell 177, 162–183. 10.1016/j.cell.2019.01.015 30901538 PMC6432948

[B124] Sun M. K. Xie W. (2012). Cell adhesion molecules in *Drosophila* synapse development and function. Sci. China Life Sci. 55, 20–26. 10.1007/s11427-012-4273-3 22314487

[B125] Tassi E. Biesova Z. Di Fiore P. P. Gutkind J. S. Wong W. T. (1999). Human JIK, a novel member of the STE20 kinase family that inhibits JNK and is negatively regulated by epidermal growth factor. J. Biol. Chem. 274, 33287–33295. 10.1074/jbc.274.47.33287 10559204

[B126] Tenedini F. M. Sáez González M. Hu C. Pedersen L. H. Petruzzi M. M. Spitzweck B.. (2019). Maintenance of cell type-specific connectivity and circuit function requires tao kinase. Nat. Commun. 10:3506. 10.1038/s41467-019-11408-1 31383864 PMC6683158

[B127] Thomas C. M. Versalovic J. (2010). Probiotics-host communication modulation of signaling pathways in the intestine. Gut Microbes 1, 1–16. 10.4161/gmic.1.3.11712 20672012 PMC2909492

[B128] Tian Y. Zhang Z. C. Han J. (2017). *Drosophila* studies on autism spectrum disorders. Neurosci. Bull. 33, 737–746. 10.1007/s12264-017-0166-6 28795356 PMC5725381

[B129] Town C. Polytechnic V. Common W. Bag P. Cruces L. Way W.. (2014). Discovery of brainwide neural-behavioral. Science 344, 386–392. 10.1126/science.1250298 24674869

[B130] Ultanir S. K. Yadav S. Hertz N. T. Oses-Prieto J. A. Claxton S. Burlingame A. L.. (2014). MST3 kinase phosphorylates TAO1/2 to enable Myosin va function in promoting spine synapse development. Neuron 84, 968–982. 10.1016/j.neuron.2014.10.025 25456499 PMC4407996

[B131] Vaccarino F. M. Smith K. M. (2009). Increased brain size in autism-what it will take to solve a mystery. Biol. Psychiatry 66, 313–315. 10.1016/j.biopsych.2009.06.013 19643218 PMC2803090

[B132] Visscher P. M. Wray N. R. Zhang Q. Sklar P. McCarthy M. I. Brown M. A.. (2017). 10 years of GWAS discovery: biology, function and translation. Am. J. Hum. Genet. 101, 5–22. 10.1016/j.ajhg.2017.06.005 28686856 PMC5501872

[B133] Wakabayashi T. Kosaka J. Oshika T. (2005). JNK inhibitory kinase is up-regulated in retinal ganglion cells after axotomy and enhances BimEL expression level in neuronal cells. J. Neurochem. 95, 526–536. 10.1111/j.1471-4159.2005.03389.x 16092929

[B134] Walsh J. J. Christoffel D. J. Heifets B. D. Ben-Dor G. A. Selimbeyoglu A. Hung L. W.. (2018). 5-HT release in nucleus accumbens rescues social deficits in mouse autism model. Nature 560, 589–594. 10.1038/s41586-018-0416-4 30089910 PMC8164568

[B135] Weigel M. Wang L. Fu M. M. (2020). Microtubule organization and dynamics in oligodendrocytes, astrocytes and microglia. Dev. Neurobiol. 1–11. 10.1002/dneu.22753 32324338

[B136] Weiss L. A. Shen Y. Korn J. M. Arking D. E. Miller D. T. Fossdal R.. (2008). Association between microdeletion and microduplication at 16p11.2 and autism. N. Engl. J. Med. 358, 667–675. 10.1056/NEJMoa075974 18184952

[B137] Wilfert A. B. Sulovari A. Turner T. N. Coe B. P. Eichler E. E. (2017). Recurrent de novo mutations in neurodevelopmental disorders: properties and clinical implications. Genome Med. 9:101. 10.1186/s13073-017-0498-x 29179772 PMC5704398

[B138] Williams D. W. Truman J. W. (2004). Mechanisms of dendritic elaboration of sensory neurons in *Drosophila*: insights from *in vivo* time lapse. J. Neurosci. 24, 1541–1550. 10.1523/JNEUROSCI.4521-03.2004 14973231 PMC6730476

[B139] Willis A. Pratt J. A. Morris B. J. (2020). BDNF and JNK signaling modulate cortical interneuron and perineuronal net development: implications for schizophrenia-linked 16p11.2 duplication syndrome. Schizophr. Bull. [Online ahead of print]. 10.1093/schbul/sbaa139 33067994 PMC8084442

[B140] Woerden G. M. Bos M. Konink C. Distel B. Trezza R. A. Shur N. E.. (2021). TAOK1 is associated with neurodevelopmental disorder and essential for neuronal maturation and cortical development. Hum. Mutat. 10.1002/humu.24176. [Online ahead of print]. 33565190 PMC8248425

[B141] Wu M. Wang S. (2008). Human TAO kinase 1 induces apoptosis in SH-SY5Y cells. Cell Biol. Int. 32, 151–156. 10.1016/j.cellbi.2007.08.006 17900936

[B142] Xie B. Fan X. Lei Y. Chen R. Wang J. Fu C.. (2016). A novel *de novo* microdeletion at 17q11.2 adjacent to NF1 gene associated with developmental delay, short stature, microcephaly and dysmorphic features. Mol. Cytogenet. 9, 9–13. 10.1186/s13039-016-0251-y 27247625 PMC4886423

[B143] Xie Y. Vessey J. P. Konecna A. Dahm R. Macchi P. Kiebler M. A. (2007). The GTP-binding protein septin 7 is critical for dendrite branching and dendritic-spine morphology. Curr. Biol. 17, 1746–1751. 10.1016/j.cub.2007.08.042 17935997

[B144] Yadav S. Oses-Prieto J. A. Peters C. J. Zhou J. Pleasure S. J. Burlingame A. L.. (2017). TAOK2 kinase mediates PSD95 stability and dendritic Spine maturation through septin7 phosphorylation. Neuron 93, 379–393. 10.1016/j.neuron.2016.12.006 28065648 PMC5267388

[B145] Yasuda S. Tanaka H. Sugiura H. Okamura K. Sakaguchi T. Tran U.. (2007). Activity-induced protocadherin arcadlin regulates dendritic spine number by triggering N-cadherin endocytosis *via* TAO2β and p38 MAP kinases. Neuron 56, 456–471. 10.1016/j.neuron.2007.08.020 17988630 PMC2424284

[B146] Ye J. Shi M. Chen W. Zhu F. Duan Q. (2020). Research advances in the molecular functions and relevant diseases of TAOKs, novel STE20 kinase family members. Curr. Pharm. Des. 26, 3122–3133. 10.2174/1381612826666200203115458 32013821

[B147] Yin Y. Donlevy S. Smolikove S. (2016). Coordination of recombination with meiotic progression in the *Caenorhabditis elegans* germline by KIN-18, a TAO Kinase that regulates the timing of MPK-1 signaling. Genetics 202, 45–59. 10.1534/genetics.115.177295 26510792 PMC4701101

[B148] Yuen R. K. C. Merico D. Bookman M. Howe J. L. Thiruvahindrapuram B. Patel R. V.. (2017). Whole genome sequencing resource identifies 18 new candidate genes for autism spectrum disorder. Nat. Neurosci. 20, 602–611. 10.1038/nn.4524 28263302 PMC5501701

[B149] Zeke A. Misheva M. Reményi A. Bogoyevitch M. A. (2016). JNK signaling: regulation and functions based on complex protein-protein partnerships. Microbiol. Mol. Biol. Rev. 80, 793–835. 10.1128/MMBR.00043-14 27466283 PMC4981676

[B151] Zhang W. Chen T. Wan T. He L. Li N. Yuan Z.. (2000). Cloning of DPK, a novel dendritic cell-derived protein kinase activating the ERK1/ERK2 and JNK/SAPK pathways. Biochem. Biophys. Res. Commun. 274, 872–879. 10.1006/bbrc.2000.3244 10924369

[B150] Zhang G. Deinhardt K. Chao M. V. Neubert T. A. (2011). Study of neurotrophin-3 signaling in primary cultured neurons using multiplex stable isotope labeling with amino acids in cell culture. J. Proteome Res. 10, 2546–2554. 10.1021/pr200016n 21370927 PMC3090507

[B152] Zhang X. Li Z. Liu Y. Gai Z. (2020). Great expectations: induced pluripotent stem cell technologies in neurodevelopmental impairments. Int. J. Med. Sci. 18, 459–473. 10.7150/ijms.51842 33390815 PMC7757149

[B153] Zhang Z. Tang Z. Ma X. Sun K. Fan L. Fang J.. (2018). TAOK1 negatively regulates IL-17-mediated signaling and inflammation. Cell. Mol. Immunol. 15, 794–802. 10.1038/cmi.2017.158 29400705 PMC6141603

[B154] Zheng X. Bei J. X. Xu H. Lee J. Chong S. A. Sim K.. (2013). The association between rare large duplication of 16p11.2 and schizophrenia in the Singaporean Chinese population. Schizophr. Res. 146, 368–369. 10.1016/j.schres.2013.02.029 23510594

[B155] Zihni C. Mitsopoulos C. Tavares I. A. Ridley A. J. Morris J. D. H. (2006). Prostate-derived sterile 20-like kinase 2 (PSK2) regulates apoptotic morphology *via* C-Jun N-terminal kinase and Rho kinase-1. J. Biol. Chem. 281, 7317–7323. 10.1074/jbc.M513769200 16407310

